# Detection of Dengue Virus and Serological Evidence of Chikungunya and Zika Virus Exposure in Patients with Acute Febrile Syndrome in Córdoba, Colombia

**DOI:** 10.3390/tropicalmed11060162

**Published:** 2026-06-17

**Authors:** Paula A. Avilés-Vergara, Dina Ricardo-Caldera, Carlos Alberto Bolívar Pineda, Eliud Daniel Pérez Vergara, Ana Carolina Negrette Oquendo, Luis Carlos Ruiz Garces, Sara Cecilia Soto-De León, Catalina Tovar-Acero

**Affiliations:** 1 Grupo de Investigación en Enfermedades Tropicales y Resistencia Bacteriana, Facultad de Ciencias de la Salud, Universidad del Sinú E.B.Z., Montería 230001, Colombia; dinaricardoc@unisinu.edu.co (D.R.-C.); anacaro9202@hotmail.com (A.C.N.O.); sarasoto@unisinu.edu.co (S.C.S.-D.L.); catalinatovar@unisinu.edu.co (C.T.-A.); 2 Programa de Doctorado en Microbiología y Salud Tropical, Universidad de Córdoba, Córdoba 230002, Colombia; luisruizg@unisinu.edu.co; 3 Programa de Medicina, Facultad de Ciencias de la Salud, Universidad del Sinú E.B.Z., Montería 230001, Colombia; carlos1620w@gmail.com (C.A.B.P.); pveliud24@gmail.com (E.D.P.V.)

**Keywords:** dengue, Zika, Chikungunya, acute febrile syndrome, seroprevalence

## Abstract

**Background/Objectives**: Arboviral diseases transmitted by *Aedes* mosquitoes, including Dengue (DENV), Zika (ZIKV), and Chikungunya (CHIKV), represent a major public health challenge in tropical regions. Their clinical similarity complicates differential diagnosis, particularly in settings of viral co-circulation, and may lead to underdiagnosis. The objective was to detect acute dengue infection and assess serological evidence of Chikungunya and Zika virus exposure among patients with acute febrile syndrome and clinical suspicion of dengue in the department of Córdoba, Colombia. **Methods**: A prospective descriptive study was conducted between 2023 and 2024 in healthcare institutions in Montería and Sahagún. Serum samples were analyzed by ELISA to detect DENV NS1 antigen, anti-CHIKV IgM, and anti-ZIKV IgG antibodies. Sociodemographic, clinical, and laboratory variables were described, and the association between prior ZIKV infection and dengue severity was assessed. **Results**: Ninety patients were included. Isolated laboratory marker detection was observed for DENV NS1 antigen in 36.7% (33/90), anti-ZIKV IgG in 30.0% (27/90), and anti-CHIKV IgM in 2.2% (2/90); combined arboviral markers were identified in 22.2% (20/90), and 8.9% (8/90) had no detectable markers. Among NS1-confirmed dengue cases (*n* = 47), 61.7% (29/47) were classified as dengue with warning signs. Anti-ZIKV IgG detection was not associated with dengue clinical classification (*p* = 0.989), although platelet counts were lower in IgG-positive cases (*p* = 0.037). **Conclusions**: The findings support laboratory-supported diagnosis and integrated acute febrile illness surveillance in Córdoba, including locally adapted vector control, in a setting of arbovirus co-circulation with overlapping laboratory markers.

## 1. Introduction

Arboviral diseases comprise a group of viral infections transmitted by hematophagous arthropods, mainly mosquitoes such as *Aedes aegypti*, predominantly adapted to urban environments, and *Aedes albopictus*, more frequently associated with rural settings [1]. In endemic regions, dengue, Zika, and chikungunya are among the most prevalent arboviral diseases. While they exhibit distinct virological characteristics, their overlapping clinical manifestations complicate differential diagnosis, particularly in settings of co-circulation.

Dengue virus (DENV) and Zika virus (ZIKV) are positive-sense single-stranded RNA viruses of the family *Flaviviridae*, *genus Orthoflavivirus* [2]. DENV consists of four antigenically distinct serotypes, enabling multiple infections throughout life; the waning of transient cross-immunity after primary infection facilitates secondary infections and sustained transmission in endemic settings. By contrast, Chikungunya virus (CHIKV) is an *Alphavirus* of the family *Togaviridae* [2]. Despite their taxonomic differences, these arboviruses share common mosquito vectors, resulting in substantial geographic and temporal co-circulation [3,4].

The widespread distribution and persistence of *Aedes aegypti* across the Americas have consolidated these arboviruses as a major public health challenge. This is reflected in the approximately tenfold increase in dengue cases reported to the World Health Organization from 2000 to 2019, underscoring the expansion and re-emergence of arboviral transmission globally [3].

In Colombia, dengue remains the arboviral disease of greatest epidemiological importance, accounting for many national arbovirus notifications in recent years. In 2020, dengue accounted for almost all reported arboviral cases, with a high clinical burden and sustained severe disease and associated mortality. Although a decline was observed in 2021, consistent with the cyclical behavior of dengue, this reduction was transient. Case numbers increased again in 2022, and by 2024 a marked rise in national incidence was documented, exceeding that of the previous four years. This endemo-epidemic pattern, characterized by interannual fluctuations and large outbreaks, reflects persistent transmission driven by the accumulation of susceptible populations and favorable environmental conditions. In Córdoba, reported incidence rates confirm its status as an endemic area, underscoring the need to strengthen surveillance and prevention strategies [5].

Zika virus circulation in Colombia has remained sustained but low following the major regional outbreak. In 2022, nearly 30,000 cases were reported in the Americas, with Brazil accounting for the highest incidence [6]. Nationally, case numbers were low and fluctuated between 2020 and 2023, with a slight increase in 2023. In Córdoba, incidence remained low, suggesting limited or underdetected transmission. By 2024, surveillance focused primarily on suspected cases associated with congenital neurological defects, potentially contributing to an underestimation of acute ZIKV circulation in the general population [5].

The notification of Chikungunya virus infection in Colombia has shown a sustained decline since 2020, suggesting reduced viral circulation and fewer active outbreaks. This trend may reflect the depletion of susceptible populations following the large outbreak in 2015, as well as strengthened surveillance, vector control, and public health response measures. Nevertheless, the persistence of clinically confirmed cases indicates residual transmission and a continued risk of re-emergence, particularly in historically endemic departments such as Córdoba, where a high disease burden and fatalities were reported during the 2015 outbreak [7,8]. These findings highlight the need to maintain epidemiological surveillance and preventive actions even in low-incidence scenarios.

Clinically, infections caused by DENV, ZIKV, and CHIKV commonly present as a nonspecific acute febrile syndrome, characterized by overlapping symptoms such as fever, headache, myalgia, arthralgia, and rash, complicating differential diagnosis. This clinical similarity, combined with the simultaneous circulation of multiple arboviruses, limits the utility of purely clinical diagnoses and contributes to the underestimation of the true burden of arboviral infections [9]. The structural similarity between dengue and Zika viruses, particularly at the level of envelope (E) glycoproteins, plays a key role in cross-immunity, whereby immune responses induced by one virus may modulate subsequent infection by the other, potentially contributing to antibody-dependent enhancement (ADE) [10]. This structural homology also underlies serological cross-reactivity among flaviviruses, which may reduce the specificity of antibody-based assays in endemic settings. Plaque reduction neutralization testing (PRNT) is considered the gold standard for differentiating flavivirus infections and minimizing cross-reactivity; however, its implementation in routine surveillance and large-scale epidemiological studies remains limited. The assay requires the handling of live viruses under appropriate biosafety conditions, sustained maintenance of cell cultures, and specialized technical expertise, making it labor-intensive, time-consuming, and costly [11]. Consequently, many epidemiological studies rely on validated serological assays despite their inherent limitations.

Serological studies remain essential for estimating prior exposure and assessing population immune status. Nevertheless, interpretation of serological findings requires caution, as IgM antibodies may persist for weeks to months after infection and IgG antibodies primarily reflect previous exposure rather than active disease. These considerations are particularly relevant in regions with sustained co-circulation of multiple arboviruses. Despite these constraints, sero-epidemiological data are critical for assessing outbreak risk, anticipating morbidity patterns, and informing prevention and control strategies in repeatedly exposed populations [12].

Despite the epidemiological importance of arboviral diseases in the Colombian Caribbean, data on acute dengue detection and serological evidence of Chikungunya and Zika virus exposure among patients with acute febrile syndrome in Córdoba remain limited. Generating local evidence is therefore essential to strengthening surveillance systems, improving etiological differentiation, and informing more effective prevention and control strategies. In this context, the objective of the present study was to detect acute dengue infection and assess serological evidence of Chikungunya and Zika virus exposure among patients with acute febrile syndrome and clinical suspicion of dengue in the department of Córdoba, Colombia.

## 2. Materials and Methods

### 2.1. Study Area and Design

A descriptive, prospective study was conducted between 2023 and 2024 in the department of Córdoba, Colombia. This region, located in the northwestern part of the country, presents eco-epidemiological conditions that favor sustained transmission of arboviral diseases. The analysis focused on Montería (the departmental capital) and Sahagún, areas of high endemicity characterized by tropical dry forest, average temperatures of approximately 28 °C, and rainfall patterns that stabilize *A. aegypti* breeding sites.

The institutional framework included healthcare centers of varying levels of complexity within the regional epidemiological surveillance network. In Montería, participant recruitment was carried out through the health centers and hospitals of the E.S.E. Vidasinú and the E.S.E. Hospital San Jerónimo, a tertiary-level referral center serving multiple areas of the department. In Sahagún, the study was conducted at the E.S.E. Hospital San Juan, which provides healthcare services to the Sabanas subregion and surrounding rural areas.

### 2.2. Samples

A passive case-finding strategy was implemented, including patients of any age and sex presenting with acute febrile syndrome (fever ≤ eight days) and clinical suspicion of dengue, according to the World Health Organization 2009 criteria [13]. Patients were recruited from the emergency departments of the participating institutions and provided written informed consent; assents were obtained for minors.

A clinical–epidemiological data collection form recorded sociodemographic and clinical information, including age, sex, place of residence, clinical manifestations, and hematological parameters.

Blood samples (approximately 5 mL) were obtained by venipuncture in gel separator tubes (Vacutainer^®^), transported at 4 °C to the Laboratorio de Investigaciones Biomédicas y Biología Molecular of the University of Sinú, Montería campus, where serum was obtained by centrifugation, aliquoted into coded Eppendorf vials, and cryopreserved at −70 °C until processing. In pediatric participants, blood volume was adjusted according to age and body weight in accordance with institutional ethical guidelines. The study protocol, including sample collection procedures, was approved by the Institutional Research Ethics Committee of the University of Sinú (Approval No. 001, 31 May 2021).

Serum samples with hemolysis, lipemia, or marked icterus were discarded due to potential interference with spectrophotometric readings. Samples with coding errors or incomplete clinical or epidemiological information were also excluded.

### 2.3. Detection of Antigens and Antibodies by Enzyme-Linked Immunosorbent Assay (ELISA)

Serum samples were analyzed using enzyme-linked immunosorbent assays (ELISA). Dengue virus NS1 antigen was detected using the Dengue NS1 Antigen DxSelect™ kit (Focus Diagnostics, Cypress, CA, USA), which has a reported sensitivity of approximately 85% and specificity of 98%, although sensitivity may be reduced in secondary dengue infections. Anti-Chikungunya virus IgM antibodies were detected using the CHIK Detect™ IgM ELISA (InBios International Inc., Seattle, WA, USA), with an estimated sensitivity of 91% and specificity of 95%; however, prolonged persistence of IgM antibodies may affect the interpretation of recent exposure. Anti-Zika virus IgG antibodies were detected using the Anti-Zika Virus ELISA IgG kit (Euroimmun, Lübeck, Germany), which reports a sensitivity of approximately 96% and specificity of 92%, although cross-reactivity with other flaviviruses, particularly dengue virus, has been described.

NS1 antigen detection was used as a marker of acute dengue infection, whereas anti-CHIKV IgM antibodies were interpreted as serological evidence of recent exposure. Anti-ZIKV IgG antibodies were used to assess prior exposure and population immune background. All assays were performed and interpreted according to the manufacturers’ instructions.

### 2.4. Statistical Analysis

Data from clinical–epidemiological forms and ELISA results were consolidated and analyzed. A descriptive analysis of sociodemographic, clinical, and laboratory variables was performed. Categorical variables were expressed as absolute frequencies and percentages, while continuous variables were summarized using medians and interquartile ranges (IQR), given their distribution. Clinical manifestations were recorded and stratified according to ELISA results. Age was categorized according to World Health Organization (WHO) criteria, and these categories were used for descriptive analyses and tabulations.

The distribution of continuous variables was assessed using the Shapiro–Wilk test. Platelet and leukocyte counts showed significant deviations from normality (platelets: W = 0.81, *p* < 0.001; leukocytes: W = 0.29, *p* < 0.001). Accordingly, non-parametric methods were used for group comparisons. Comparisons of hematological parameters between dengue cases with and without warning signs were conducted using the Mann–Whitney U test for continuous variables (platelet and leukocyte counts). Differences in the frequency of thrombocytopenia between groups were evaluated using Fisher’s exact test.

The potential association between previous Zika virus infection (anti-ZIKV IgG seropositivity) and dengue clinical severity was explored using contingency tables and bivariate analyses (Pearson’s chi-square or Fisher’s exact test, as appropriate). Additionally, platelet counts were compared according to anti-ZIKV IgG serostatus using the Mann–Whitney U test.

To account for potential confounding, multivariate analyses were performed. A binary logistic regression model was used to evaluate the association between prior Zika virus infection (anti-ZIKV IgG seropositivity) and dengue clinical severity (with vs. without warning signs), adjusting for age (included as a continuous variable) and sex. Only patients with confirmed dengue classification (with or without warning signs) were included in this analysis, and cases categorized as “not applicable” were excluded.

Additionally, the relationship between anti-ZIKV IgG serostatus and platelet counts was explored using linear regression models. Platelet counts were log-transformed to approximate normality, and models were adjusted for age and sex using robust standard errors. Due to the sample size, models were kept parsimonious to avoid overfitting. A *p*-value < 0.05 was considered statistically significant.

All statistical analyses were performed using Stata version 14 (StataCorp, College Station, TX, USA). Geographic maps were created using QGIS version 3.28.4 (QGIS Development Team).

## 3. Results

A total of 150 samples were collected; 90 met the established inclusion criteria and were included in the final analysis. Serum samples were analyzed using enzyme-linked immunosorbent assays (ELISA) to detect DENV NS1 antigen as a marker of acute dengue infection, and anti-CHIKV IgM and anti-ZIKV IgG antibodies as indicators of recent or prior arboviral exposure.

Patients’ ages ranged from 3 to 86 years, with a mean age of 17.3 ± 13.9 years (median: 14 years; interquartile range [IQR]: 10–17 years), reflecting the predominance of children and adolescents in the study population. Of the 90 participants, 55.56% (50/90) were male, and 44.44% (40/90) were female.

**Figure 1:**
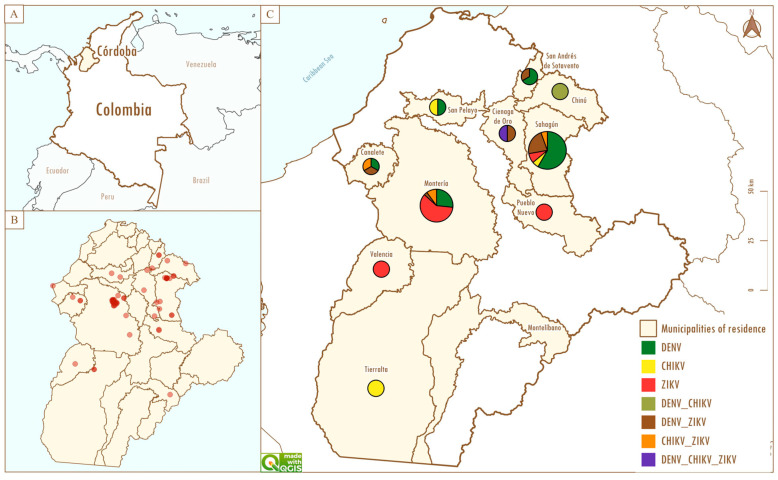
Spatial distribution and seroprevalence of dengue virus and other arboviruses in the department of Córdoba, 2023–2024: (**A**) Geographic location of the study area. (**B**) Spatial distribution of patients with acute febrile syndrome included in the study. (**C**) Municipal circulation of Dengue virus (DENV), Zika virus (ZIKV), and Chikungunya virus (CHIKV) based on ELISA results; pie charts represent the proportion of seropositive patients for each arbovirus within each municipality. Circle size is proportional to the total number of patients sampled.

Cases originated from 11 municipalities in Córdoba, mainly Sahagún, Chinú, and San Andrés de Sotavento (Subregión Sabana), which accounted for 51.11% (46/90) of cases. Montería contributed 34.44% (31/90), and the remaining 14.44% (13/90) was contributed by Canalete, Ciénaga de Oro, Montelíbano, Pueblo Nuevo, San Pelayo, Tierralta, and Valencia (Table 1, Figure 1B).

Among the 90 patients included in the study, isolated laboratory marker detection was observed for DENV NS1 antigen in 36.7% (33/90), anti-ZIKV IgG in 30.0% (27/90), and anti-CHIKV IgM in 2.2% (2/90). Combined arboviral markers (i.e., detection of ≥2 markers among DENV NS1, anti-CHIKV IgM, and anti-ZIKV IgG) were identified in 22.2% (20/90) of patients, while 8.9% (8/90) had no detectable markers (Table 1).

Figure 1 summarizes the distribution of arboviral laboratory marker combinations in the study population. Combined arboviral markers were identified in 22.2% (20/90) of patients. The most frequent pattern was co-detection of dengue NS1 antigen and anti-ZIKV IgG antibodies (*n* = 12), followed by co-detection of anti-CHIKV IgM and anti-ZIKV IgG (*n* = 6). Less frequent combinations included dengue NS1 antigen with anti-CHIKV IgM (*n* = 1) and the simultaneous detection of dengue NS1 antigen, anti-CHIKV IgM, and anti-ZIKV IgG (*n* = 1). These combinations should be interpreted as overlapping laboratory markers in a co-circulation setting rather than definitive evidence of simultaneous acute infections, given the antibody-based nature of IgM/IgG testing.

### 3.1. Acute Dengue Infection (NS1 Detection)

Among patients with acute dengue infection confirmed by NS1 antigen detection (*n* = 47), males accounted for 57.4% of cases. The most affected age groups were children and adolescents, which together represented 89.4% (42/47) of dengue cases. Most infections were reported from the Sabana subregion, which accounted for 70.2% (33/47) of cases. Within NS1-confirmed dengue cases, isolated NS1 positivity accounted for 70.2% (33/47), while 29.8% (14/47) had at least one additional arboviral marker. Anti-ZIKV IgG was co-detected in 27.7% (13/47) and anti-CHIKV IgM in 4.3% (2/47); one case showed both antibodies detected together with NS1 (triple-marker detection), indicating overlapping laboratory markers in a co-circulation setting rather than definitive evidence of simultaneous acute infections (Table 2).

According to the WHO clinical classification, 38.3% (18/47) of cases were categorized as dengue without warning signs, whereas 61.7% (29/47) were classified as dengue with warning signs (Table 2). Fever was present in all patients and remained the most consistent clinical manifestation. Headache occurred in 76.6% of cases and was slightly more frequent among patients without warning signs. Myalgia and arthralgia were observed in 29.8% and 31.9% of patients, respectively, and were more frequent in the dengue without warning signs group. Rash was identified in 38.3% of cases and was more frequent among patients with warning signs (Table 2).

Symptoms such as nausea, loss of appetite, dizziness, retro-orbital pain, fatigue, hepatomegaly, mucosal bleeding, and edema were reported in fewer than 15% of cases overall. In contrast, the most common warning signs were severe abdominal pain (44.7%), persistent vomiting (46.8%), and diarrhea (42.6%). Regarding hematological findings, leukopenia was observed in 51.1% of patients and was more frequent in cases with warning signs (62.1%) than in those without (33.3%). Thrombocytopenia was detected in 44.7% of cases and occurred in both clinical groups, indicating that reduced platelet counts can be observed across the clinical spectrum of dengue (Table 2).

Normality of continuous variables was assessed using the Shapiro–Wilk test. Platelet and leukocyte counts showed significant deviations from normality (platelets: W = 0.81, *p* < 0.001; leukocytes: W = 0.29, *p* < 0.001) and were therefore analyzed using non-parametric methods. When comparing hematological parameters between dengue cases with and without warning signs, no statistically significant differences were observed. Platelet counts did not differ significantly between the two groups (Mann–Whitney U test; *p* = 0.36), nor did leukocyte counts (Mann–Whitney U test; *p* = 0.94). Likewise, although thrombocytopenia was more frequent among cases with warning signs (55.2%, n = 16) compared with cases without warning signs (27.8%, *n* = 5), this difference did not reach statistical significance (Fisher’s exact test; *p* = 0.136). In contrast, when platelet counts were analyzed according to anti-ZIKV IgG serostatus, patients with positive IgG showed significantly lower values compared with IgG-negative patients (median: 89,500; IQR: 61,000 vs. 132,000; IQR: 105,500; Mann–Whitney U test; *p* = 0.037).

To further explore these associations, multivariate analyses were performed. In logistic regression models adjusted for age and sex, no significant association was observed between anti-ZIKV IgG seropositivity and dengue clinical severity (adjusted OR = 1.16, 95% CI: 0.29–4.56, *p* = 0.832). Similarly, in linear regression models using log-transformed platelet counts, no significant association was found between anti-ZIKV IgG serostatus and platelet levels after adjustment for age and sex (β = −0.18, 95% CI: −0.44 to 0.08, *p* = 0.168).

### 3.2. Detection of Anti-CHIKV IgM Antibodies

Anti-CHIKV IgM antibodies were detected in 11.1% (10/90) of the study population. The present section describes the 8 patients without dengue NS1 antigen detected (8.9%, 8/90), while the remaining two anti-CHIKV IgM-positive patients were included among NS1-confirmed dengue cases (Table 2) and are reflected in the combined-marker patterns shown in Figure 1 (Table 1; Figure 1).

Among these 8 patients, females accounted for 62.5% of cases, and most individuals were children and adolescents (≤18 years), who together represented 75% of cases. Patients originated from the municipalities of Sahagún, Montería, and Canalete (Table 1, Figure 1). Regarding clinical findings, fever and headache were reported in all individuals with anti-CHIKV IgM detection, followed by arthralgia, vomiting, and diarrhea. Other manifestations, such as rash, myalgia, loss of appetite, nausea, and dizziness, were reported less frequently.

Regarding laboratory parameters, thrombocytopenia was observed in 87.5% of patients, while leukocytosis was detected in 25%; these hematological alterations were present in a subset of patients with anti-CHIKV IgM detection (Table 3).

### 3.3. Detection of Anti-ZIKV IgG Antibodies

Anti-ZIKV IgG antibodies were detected in 30% (27/90) of the study population, indicating serological evidence of prior exposure to Zika virus. Among these individuals, a slight predominance of males was observed (55.6%). Most patients belonged to the childhood and adolescent age groups, which together accounted for 74.1% of cases. Regarding geographic origin, the largest proportion of patients came from the Sinú Medio subregion (66.7%), followed by Alto Sinú (14.8%), Sabana (11.1%), and San Jorge (7.4%) (Table 1, Figure 1).

In this group, fever was reported in all patients, followed by headache (63.0%), severe abdominal pain (44.4%), persistent vomiting (44.4%), and diarrhea (40.7%). Rash and arthralgia (33.3% each), myalgia (25.9%), and somnolence or irritability (22.2%) were also observed, while dizziness, loss of appetite, and nausea were less frequent. Laboratory findings included thrombocytopenia (51.8%) and leukopenia (48.1%).

These findings reflect the acute febrile illness presentation among patients with isolated anti-ZIKV IgG detection and should be interpreted cautiously, as IgG positivity primarily indicates prior exposure rather than active infection at the time of sampling (Table 3).

The association between anti-ZIKV IgG detection and dengue clinical classification was evaluated among NS1-confirmed dengue cases (*n* = 47). The proportion of cases with warning signs was similar among patients with anti-ZIKV IgG detected and those without detectable IgG antibodies. Bivariate analysis showed no evidence of an association between anti-ZIKV IgG detection and dengue clinical classification in this sample (Pearson’s chi-square test, *p* = 0.989; Fisher’s exact test, *p* = 1.000) (Table 4).

## 4. Discussion

The results of this study showed that 36.7% of acute febrile syndrome cases were classified as acute dengue infection based on NS1 antigen detection. Although the initial clinical suspicion was directed exclusively toward dengue, anti-CHIKV IgM antibodies were detected in 11.1% of patients, providing serological evidence of recent exposure and supporting the likelihood of ongoing Chikungunya virus circulation, which may be underrecognized when CHIKV testing is not routinely performed in clinical practice. Co-detection of dengue NS1 antigen and anti-CHIKV IgM was observed in two patients and should be interpreted as overlapping laboratory markers rather than as confirmed coinfection in the absence of molecular testing.

In addition, anti-ZIKV IgG antibodies were detected in 30% of patients, indicating serological evidence of prior exposure to Zika virus (or other circulating flaviviruses in endemic settings). Notably, 13.3% of cases showed simultaneous detection of dengue NS1 antigen and anti-ZIKV IgG, suggesting acute dengue infection in individuals with pre-existing flavivirus antibodies. Overall, these findings reinforce that dengue often remains the predominant clinical diagnosis, potentially masking other arboviral exposures and relevant immunological backgrounds when broad arboviral screening is not routinely implemented. This pattern is consistent with national reports describing arbovirus co-circulation as a diagnostic challenge, in which overlapping infections or exposures may be underrecognized in the absence of confirmatory testing [14,15].

Regarding the sociodemographic profile, a predominance of male patients (55.56%) was observed, particularly among NS1-confirmed dengue cases (57.4%), consistent with national reports attributing higher prevalence in males from hyperendemic regions to behavioral and environmental exposure, including outdoor activities during peak vector activity [15].

Most cases occurred among adolescents (46.67%) and children (31.11%), consistent with Colombian studies identifying individuals aged 5–14 years as the group with the highest frequency of acute arboviral infections, possibly related to the relative immaturity of the immune response in endemic settings [16]. The lower frequency observed in adults, together with the detection of anti-ZIKV IgG antibodies in 30% of patients, suggests a pattern of prior flavivirus exposure that may contribute to shifting the burden of acute febrile illness toward younger age groups [10]. In addition, the co-circulation of multiple arboviruses in the Sinú Medio and Sabana subregions further highlights the diagnostic complexity of hyperendemic settings, where dengue often functions as an “umbrella” diagnosis that may obscure other clinically similar arboviral exposures [15,17].

Geographically, the Sabana subregion concentrated the highest proportion of patients with acute dengue infection and anti-CHIKV IgM detection, whereas the Sinú Medio subregion showed the highest frequency of anti-ZIKV IgG detection. These differences may reflect variation in viral circulation, prior population exposure, and socioenvironmental conditions that favor vector transmission. In Córdoba, high multidimensional poverty (36.7%) and persistent deficiencies in access to potable water, sewerage, and waste collection represent structural determinants that should be considered in the design and implementation of more effective prevention and control strategies for dengue and other arboviral diseases [16].

The clinical and laboratory findings observed in this study are consistent with reports from other arbovirus-endemic settings. Fever was the most frequent manifestation among patients with acute dengue infection, reinforcing its value as the cardinal symptom in the clinical presentation of arboviral febrile illness [18,19]. Among the warning signs, severe abdominal pain, persistent vomiting, and diarrhea were the most prominent clinical manifestations, supporting their relevance in identifying patients at greater risk of clinical deterioration. Similarly, leukopenia and thrombocytopenia were frequent laboratory abnormalities, which is consistent with their recognized value as markers of disease progression in dengue [20], whereas previous studies in Córdoba reported mainly outpatient and uncomplicated cases [21]. Given the hospital-based recruitment (emergency/referral settings), the high proportion of cases with warning signs should be interpreted as likely influenced by the selection of more severe presentations. These findings should therefore be interpreted with caution, particularly given the limited sample size, but they remain clinically relevant, highlighting the importance of combining clinical warning signs with laboratory parameters to support timely recognition and management of potentially severe dengue cases.

An important consideration when interpreting our laboratory findings is the diagnostic meaning and limitations of the markers assessed. NS1 antigen detection supports the identification of acute dengue infection, whereas antibody-based assays reflect host immune responses and should be interpreted with caution in endemic settings. Anti-CHIKV IgM antibodies may persist for weeks to months after infection [22]. Therefore, IgM positivity should be interpreted as evidence of recent exposure rather than definitive confirmation of active infection in the absence of molecular testing or paired acute–convalescent serology. Likewise, isolated anti-ZIKV IgG positivity primarily indicates prior exposure and does not support attribution of the current febrile episode to active Zika virus infection.

Interpretation is further complicated by the well-recognized serological cross-reactivity among flaviviruses, particularly between DENV and ZIKV, largely driven by structural homology in envelope proteins. As a result, antibody-based assays may overestimate virus-specific exposure in areas with repeated flavivirus circulation [23].

Plaque reduction neutralization testing (PRNT) remains the reference method for improving specificity and differentiating flavivirus infections; however, its use is often limited to reference laboratories because it requires live virus, appropriate biosafety conditions, and cell culture infrastructure, and is labor-intensive and time-consuming. Consequently, epidemiological studies frequently rely on validated serological assays despite their known constraints, underscoring the importance of cautious, transparent interpretation of antibody findings in co-circulation settings [11].

Among patients with anti-CHIKV IgM detection, thrombocytopenia and leukopenia were frequent, suggesting that hematological involvement may be clinically relevant beyond the classically described arthralgia [20,24]. In patients with isolated anti-ZIKV IgG detection, headache, abdominal pain, vomiting, leukopenia, and thrombocytopenia were also observed. Because IgG indicates prior exposure rather than acute infection, these findings should be interpreted as the acute febrile illness presentation in patients with evidence of prior flavivirus exposure and may reflect other etiologies circulating locally. Previous studies in Córdoba have documented the circulation of several pathogens associated with febrile illness, including Mayaro virus (MAYV), Venezuelan equine encephalitis virus (VEEV), West Nile virus (WNV), and, to a lesser extent, *Leptospira* spp. and *Rickettsia* spp. [25,26].

Furthermore, the recent documentation of *Aedes albopictus* in the department represents an epidemiologically relevant finding, given its recognized role as a competent or potential vector for multiple arboviruses [27]. The establishment of this species may facilitate the circulation of emerging and re-emerging arboviruses, broadening the spectrum of pathogens responsible for acute febrile syndromes with dengue-like presentations. Importantly, the presence and potential expansion of *A. albopictus* in Córdoba may contribute—together with *Aedes aegypti* and prevailing socioenvironmental conditions—to sustaining arbovirus transmission in the department. In line with our findings, including the frequent detection of combined arboviral markers, the establishment of *A. albopictus* may increase the likelihood of arbovirus co-circulation and broaden dengue-like acute febrile presentations in routine clinical settings. Because these syndromes are already clinically difficult to distinguish, this context further emphasizes the need for laboratory-supported diagnosis and integrated surveillance rather than reliance on clinical criteria alone. From a public health perspective, this supports strengthening integrated entomological and epidemiological surveillance, monitoring the geographic distribution and seasonal dynamics of *A. albopictus*, and adapting vector control strategies to address multiple vectors and diverse breeding habitats. Such measures are particularly relevant in Córdoba, where structural determinants and environmental conditions may favor sustained vector proliferation, highlighting the importance of targeted community-based interventions and timely outbreak preparedness.

Overall, the clinical overlap among DENV, CHIKV, and ZIKV limits differential diagnosis based solely on clinical criteria, highlighting the need for serological support and specific laboratory testing. The observation of thrombocytopenia and leukopenia among patients with anti-CHIKV IgM detected and/or isolated anti-ZIKV IgG detected, although classically associated with dengue, indicates that these alterations are not exclusive to a single arbovirus. This underscores the importance of maintaining a broad clinical suspicion and implementing complementary diagnostic strategies, such as NS1 testing during the first 72 h of dengue suspicion or multiplex PCR in severe or atypical cases, particularly in regions with arbovirus co-circulation [12].

In this study, anti-ZIKV IgG detection (as serological evidence consistent with prior flavivirus exposure) was not associated with dengue clinical classification among NS1-confirmed dengue cases. Patients with anti-ZIKV IgG detected showed a clinical course similar to those without detectable IgG, predominantly presenting a typical dengue-like illness. These findings are consistent with previous studies reporting that overlapping arboviral exposures frequently resemble classical dengue without a consistent increase in severity [28]. However, recent evidence is heterogeneous. A study evaluating the influence of prior ZIKV infection on subsequent dengue episodes reported differences in dengue outcomes among individuals with documented prior ZIKV exposure, underscoring that the clinical impact of pre-existing ZIKV immunity may vary across settings and study designs [29]. In addition, a long-term cohort study showed that primary ZIKV exposure was associated with an increased risk of symptomatic dengue for DENV serotypes 2–4, but not serotype 1, suggesting that the effect of prior ZIKV immunity may depend on the circulating serotype and the outcome definition (symptomatic infection vs. clinical severity) [30]. Interpretation of our findings should therefore remain cautious because anti-ZIKV IgG assays may have limited specificity in settings with repeated flavivirus circulation, and cross-reactivity with dengue antibodies may contribute to IgG detection [31]. Moreover, the hospital-based design and sample size may limit the power to detect modest associations.

Lower platelet counts observed among NS1-confirmed dengue cases with anti-ZIKV IgG detected may reflect immunological interactions described among flaviviruses, including modulation by pre-existing heterologous antibodies (e.g., mechanisms discussed under antibody-dependent enhancement, ADE) [18,32]. Nonetheless, in this sample, anti-ZIKV IgG detection was not associated with a higher proportion of dengue cases with warning signs, suggesting that any such interactions—if present—may influence hematological parameters without translating into more clinically severe disease. These findings should be interpreted with caution given the cross-sectional design, the sample size, and the absence of molecular confirmation or functional neutralization assays.

Overall, these findings highlight that acute febrile illness in endemic regions such as Córdoba occurs within a complex co-circulation context characterized by overlapping clinical manifestations. The detection of acute dengue markers together with frequent antibody evidence of prior flavivirus exposure underscores the limitations of symptom-based diagnosis and supports the need for laboratory-supported etiologic differentiation and expanded surveillance approaches to better characterize arboviral circulation and other potential etiologies.

These results reinforce the need to strengthen laboratory diagnostics, expand surveillance panels, and consolidate integrated vector control and epidemiological surveillance strategies. Interpretation of our findings is limited by the relatively small sample size, hospital-based design, and the use of antibody-based assays without molecular confirmation or neutralization testing, which do not precisely define infection timing and may have reduced the power to detect subtle associations. Accordingly, negative findings should be interpreted with caution. The hospital-based nature of the cohort may also limit generalizability to the broader Córdoba population. Nevertheless, these results provide relevant preliminary insights in an endemic setting and should be considered hypothesis-generating. Further studies using molecular diagnostics, confirmatory serology (e.g., neutralization assays), longitudinal designs, and multicenter approaches are warranted to better characterize arboviral interactions and their clinical implications.

## 5. Conclusions

This study provides local evidence that acute febrile illness in Córdoba occurs in a context of arbovirus co-circulation, where dengue is frequently detected and overlapping laboratory markers are also observed. These findings support strengthening laboratory-supported diagnosis in routine care, as symptom-based differentiation is insufficient in hyperendemic settings; early NS1 testing for suspected dengue and broader etiologic consideration in warning-sign or atypical cases may improve the timeliness of clinical management.

From a public health perspective, the results support integrating acute febrile illness surveillance with expanded arboviral testing where feasible, alongside sustained entomological monitoring and vector control adapted to local eco-epidemiological conditions. In Córdoba, surveillance should prioritize early outbreak detection and reinforce referral pathways for patients with warning signs.

## Figures and Tables

**Table 1:** Distribution of sociodemographic characteristics and arboviral laboratory markers among patients with acute febrile syndrome in Córdoba, Colombia.

Variable	Category	Acute DENV Infection (NS1)	ZIKV IgG Detected	CHIKV IgM Detected	Combined Arboviral Markers	Negative	Total
		*n* (%)	*n* (%)	*n* (%)	*n* (%)	*n* (%)	*n* (%)
**Total patients**		33 (36.7)	27 (30.0)	2 (2.2)	20 (22.2)	8 (8.9)	90 (100)
**Sex**	Male	21 (63.64)	15 (55.56)	1 (50)	8 (40.0)	5 (62.5)	50 (55.56)
	Female	12 (36.36)	12 (44.44)	1 (50)	12 (60.0)	3 (37.5)	40 (44.44)
**Age group (years)**	Childhood (0–11)	14 (42.42)	5 (18.52)	–	8 (40.0)	1 (12.5)	28 (31.11)
	Adolescence (12–18)	16 (48.48)	15 (55.56)	–	8 (40.0)	3 (37.5)	42 (46.67)
	Young adults (19–26)	2 (6.06)	3 (11.11)	2 (100)	2 (10.0)	1 (12.5)	10 (11.11)
	Adults (27–59)	1 (3.03)	3 (11.11)	–	1 (5.0)	2 (25.0)	7 (7.78)
	Older adults (≥60)	–	1 (3.70)	–	1 (5.0)	1 (12.5)	3 (3.33)
**Subregion of origin**	Alto Sinú	–	3 (11.11)	–	–	–	3 (3.33)
	Costanera	1 (3.03)	–	–	2 (10.0)	–	3 (3.33)
	Sabana	23 (69.70)	3 (11.11)	2 (100)	12 (60.0)	6 (75.0)	46 (51.11)
	San Jorge	–	2 (7.41)	–	–	1 (12.5)	3 (3.33)
	Sinú Medio	9 (27.27)	19 (70.37)	–	6 (30.0)	1 (12.5)	35 (38.89)

Notes: The symbol “–” indicates that no cases were observed in that category. “Combined arboviral markers” correspond to patients in whom more than one arboviral laboratory marker was detected. Percentages represent the proportion within each diagnostic category.

**Table 2:** Sociodemographic, clinical, and laboratory characteristics of NS1-confirmed dengue cases according to WHO 2009 classification.

Variable	Category	Dengue Without Warning Signs (*n* = 18)	Dengue with Warning Signs (*n* = 29)	Total (*n* = 47)
		*n* (%)	*n* (%)	*n* (%)
Sex	Male	8 (44.4)	19 (65.5)	27 (57.4)
	Female	10 (55.6)	10 (34.5)	20 (42.6)
Age group (years)	Childhood (0–11)	7 (38.9)	14 (48.3)	21 (44.7)
	Adolescence (12–18)	9 (50)	12 (41.4)	21 (44.7)
	Youth (19–26)	1 (5.6)	2 (6.9)	3 (6.4)
	Adulthood (27–59)	1 (5.6)	1 (3.4)	2 (4.3)
Subregion of origin	Costanera	2 (11.1)	–	2 (4.3)
	Sabana	11 (61.1)	22 (75.9)	33 (70.2)
	Sinú Medio	5 (27.8)	7 (24.1)	12 (25.5)
Clinical manifestations	Fever	18 (100)	29 (100)	47 (100)
	Nausea	1 (5.6)	–	1 (2.1)
	Loss of appetite	1 (5.6)	1 (3.4)	2 (4.3)
	Headache	14 (77.8)	22 (75.9)	36 (76.6)
	Myalgia	6 (33.3)	8 (27.6)	14 (29.8)
	Arthralgia	7 (38.9)	8 (27.6)	15 (31.9)
	Rash	4 (22.2)	14 (48.3)	18 (38.3)
	Dizziness	1 (5.6)	1 (3.4)	2 (4.3)
	Retro-orbital pain	1 (5.6)	5 (17.2)	6 (12.8)
	Fatigue	1 (5.6)	–	1 (2.1)
	Severe abdominal pain	–	21 (72.4)	21 (44.7)
	Persistent vomiting	–	22 (75.9)	22 (46.8)
	Diarrhea	–	20 (69)	20 (42.6)
	Somnolence/irritability	–	5 (17.2)	5 (10.6)
	Hepatomegaly	–	1 (3.4)	1 (2.1)
	Mucosal bleeding	–	5 (17.2)	5 (10.6)
	Edema	–	1 (3.4)	1 (2.1)
Laboratory findings	Leukopenia (WBC < 4500 cells/µL)	6 (33.3)	18 (62.1)	24 (51.1)
	Thrombocytopenia (platelets < 100,000/µL)	5 (27.8)	16 (55.2)	21 (44.7)
	Anti-CHIKV IgM detected	1 (5.6)	1 (3.4)	2 (4.3)
	Anti-ZIKV IgG detected	5 (27.8)	8 (27.6)	13 (27.7)

Notes: The symbol “–” indicates that no cases were observed. Anti-CHIKV IgM and anti-ZIKV IgG rows are not mutually exclusive (one patient had co-detection of both antibodies together with NS1).

**Table 3:** Signs, symptoms, and hematological findings according to arboviral laboratory marker categories.

Signs and Symptoms		DENV NS1 (*n* = 33)	ZIKV IgG Detected (*n* = 27)	CHIKV IgM Detected (*n* = 2)	Combined Arboviral Markers (*n* = 20)	Negatives (*n* = 8)	Total (*n* = 90)
		*n* (%)	*n* (%)	*n* (%)	*n* (%)	*n* (%)	*n* (%)
Neurological	Somnolence and irritability	4 (12.1)	3 (11.1)	–	1 (5.0)	–	8 (8.9)
	Headache	26 (78.8)	19 (70.4)	2 (100)	16 (80.0)	8 (100)	71 (78.9)
	Dizziness	2 (6.1)	3 (11.1)	1 (50.0)	–	1 (12.5)	7 (7.8)
	Retro-orbital pain	3 (9.1)	1 (3.7)	–	4 (20.0)	–	8 (8.9)
Gastrointestinal	Nausea	1 (3.0)	5 (18.5)	1 (50.0)	–	2 (25.0)	9 (10.0)
	Loss of appetite	2 (6.1)	1 (3.7)	1 (50.0)	–	1 (12.5)	5 (5.6)
	Persistent vomiting	15 (45.5)	14 (51.9)	–	9 (45.0)	4 (50.0)	42 (46.7)
	Diarrhea	17 (51.5)	9 (33.3)	–	5 (25.0)	3 (37.5)	34 (37.8)
	Abdominal pain	17 (51.5)	13 (48.1)	–	5 (25.0)	6 (75.0)	41 (45.6)
	Hepatomegaly	1 (3.0)	1 (3.7)	–	–	–	2 (2.2)
Musculoskeletal	Myalgia	11 (33.3)	8 (29.6)	–	4 (20.0)	3 (37.5)	26 (28.9)
	Arthralgia	10 (30.3)	8 (29.6)	–	9 (45.0)	4 (50.0)	31 (34.4)
Systemic	Fatigue	1 (3.0)	2 (7.4)	1 (50.0)	–	1 (12.5)	5 (5.6)
	Fever	33 (100)	27 (100)	2 (100)	20 (100)	8 (100)	90 (100)
	Hypothermia	–	1 (3.7)	–	–	–	1 (1.1)
	Hypertension	–	1 (3.7)	–	–	–	1 (1.1)
	Edema	1 (3.0)	1 (3.7)	–	–	–	2 (2.2)
Cutaneous	Rash	15 (45.5)	8 (29.6)	–	5 (25.0)	5 (62.5)	33 (36.7)
	Exanthema	–	2 (7.4)	–	1 (5.0)	–	3 (3.3)
Hematological	Mucosal bleeding	4 (12.1)	3 (11.1)	–	1 (5.0)	–	8 (8.9)
	Thrombocytopenia (platelets < 100,000/µL)	12 (36.4)	14 (51.9)	2 (100)	18 (90.0)	2 (25.0)	48 (53.3)
	Leukopenia (WBC < 4500/µL)	16 (48.5)	13 (48.1)	1 (50.0)	18 (90.0)	4 (50.0)	52 (57.8)
	Leukocytosis (WBC > 11,000/µL)	–	–	1 (50.0)	2 (10.0)	–	3 (3.3)

Notes: “–” indicates that no cases were observed in that category. Percentages are calculated within each category. “Combined arboviral markers” indicates detection of ≥2 markers among DENV NS1, anti-CHIKV IgM, and anti-ZIKV IgG. Detailed characterization of NS1-confirmed dengue cases (*n* = 47) is presented in Table 2.

**Table 4:** Association between anti-ZIKV IgG detection and dengue clinical classification among NS1-confirmed dengue cases.

Anti-ZIKV IgG	Dengue Without Warning Signs *n* (%)	Dengue with Warning Signs *n* (%)	Total *n* (%)	*p* Valor
Detected	5 (27.8)	8 (27.6)	13 (27.7)	-
Not detected	13 (72.2)	21 (72.4)	34 (72.3)	
Total	18 (100)	29 (100)	47 (100)	
Pearson’s chi-square test				0.989
Fisher’s exact test				1.000

## Data Availability

The data presented in this study are available in the article. Additional data are available from the corresponding author upon reasonable request.

## Abbreviations

The following abbreviations are used in this manuscript:

DENV	Dengue virus
CHIKV	Chikungunya virus
ZIKV	Zika virus
MAYV	Mayaro virus
VEEV	Venezuelan equine encephalitis virus
WNV	West Nile virus
